# Analysis and experimental validation of necroptosis-related molecular classification, immune signature and feature genes in Alzheimer’s disease

**DOI:** 10.1007/s10495-024-01943-8

**Published:** 2024-03-13

**Authors:** Piaopiao Lian, Xing Cai, Xiaoman Yang, Zhuoran Ma, Cailin Wang, Ke Liu, Yi Wu, Xuebing Cao, Yan Xu

**Affiliations:** 1 Department of Neurology, Union Hospital, Tongji Medical College, Huazhong University of Science and Technology, Wuhan, China; 2 Department of Oncology, Union Hospital, Tongji Medical College, Huazhong University of Science and Technology, Wuhan, China; 3 Department of Neurology, Renmin Hospital of Wuhan University, Wuhan, China

**Keywords:** Alzheimer’s disease, Necroptosis, Bioinformatic analysis, Immune infiltration, Machine learning

## Abstract

Necroptosis, a programmed cell death pathway, has been demonstrated to be activated in Alzheimer’s disease (AD). However, the precise role of necroptosis and its correlation with immune cell infiltration in AD remains unclear. In this study, we conducted non-negative matrix factorization clustering analysis to identify three subtypes of AD based on necroptosis-relevant genes. Notably, these subtypes exhibited varying necroptosis scores, clinical characteristics and immune infiltration signatures. Cluster B, characterized by high necroptosis scores, showed higher immune cell infiltration and was associated with a more severe pathology, potentially representing a high-risk subgroup. To identify potential biomarkers for AD within cluster B, we employed two machine learning algorithms: the least absolute shrinkage and selection operator regression and Random Forest. Subsequently, we identified eight feature genes (CARTPT, KLHL35, NRN1, NT5DC3, PCYOX1L, RHOQ, SLC6A12, and SLC38A2) that were utilized to develop a diagnosis model with remarkable predictive capacity for AD. Moreover, we conducted validation using bulk RNA-seq, single-nucleus RNA-seq, and in vivo experiments to confirm the expression of these feature genes. In summary, our study identified a novel necroptosis-related subtype of AD and eight diagnostic biomarkers, explored the roles of necroptosis in AD progression and shed new light for the clinical diagnosis and treatment of this disease.

**Supplementary Information:**

The online version contains supplementary material available at 10.1007/s10495-024-01943-8.

## Introduction

Alzheimer’s disease (AD) is a progressive neurodegenerative disease and represents the leading cause of dementia [1]. It manifests as a gradual decline in cognitive function, memory loss, and significant impairment of daily functioning. The global incidence of AD has been steadily rising, posing a continuous burden [2]. Unfortunately, there are currently no effective treatments available to cure or halt the progression of this disease. Neuropathologically, AD is characterized by the extracellular amyloid-β (Aβ) deposition, the intracellular hyperphosphorylated Tau (p-Tau) leading to the development of neurofibrillary tangles (NFTs), severe neuronal loss, and neuroinflammation. While the precise pathogenesis of AD remains incompletely understood. Recent studies suggested that necroptosis may play a significant role in cell death processes of AD [3–5].

Damage and loss of nerve cells are prominent features in the pathology of AD. However, the specific mechanisms underlying neuronal death are not fully elucidated. Recent research has identified the activation of necroptosis, a programmed cell death pathway, in AD [5]. This process involves the oligomerization of mixed lineage kinase domain-like protein (MLKL), which undergoes phosphorylated by the receptor-interacting serine–threonine protein kinase 1 (RIPK1) and RIPK3 [6]. Subsequently, oligomerized MLKL translocates to the plasma membrane, leading to apoptosis of cells [7, 8]. Notably, elevated expression of RIPK1 and MLKL has been detected in the temporal gyrus of AD patients [5]. Animal experiments using TauP301S mice have further demonstrated that inhibition of necroptosis significantly attenuated behavioral abnormalities and mitigated excessive neuroinflammation [9]. Inflammation and immune dysregulation are also fundamental features in the pathophysiology of Alzheimer’s disease, contributing to its progression. Nevertheless, the precise role of necroptosis in the etiology of Alzheimer’s disease requires clarification.

**Fig. 1:**
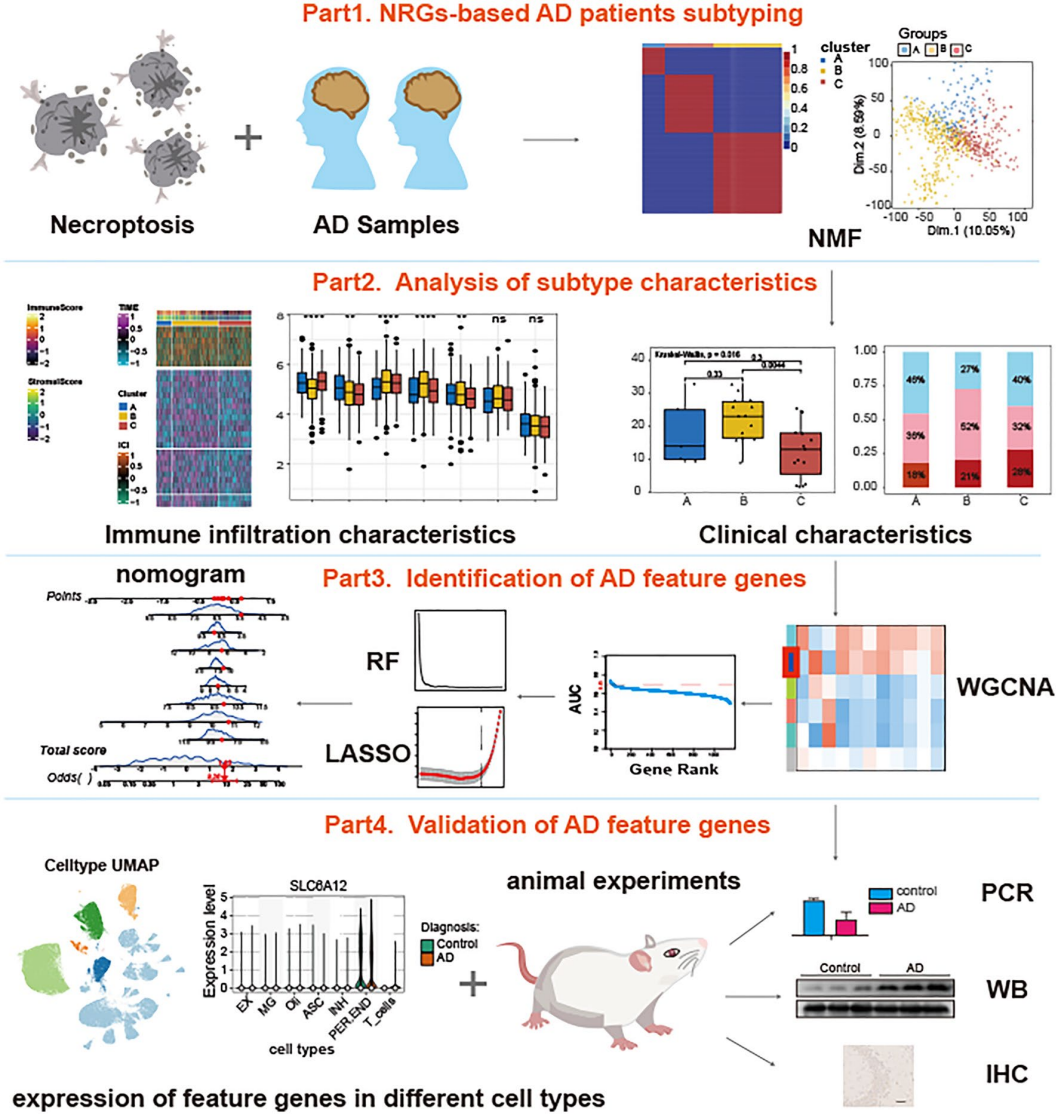
Flowchart of the study. Part 1: NRGs-based AD patients subtyping. After preprocessing of AD datasets, we classified the AD patients into distinct subtypes based on the 159 NRGs using NMF. The ADs were classified into three subtypes. Part 2: Analysis of subtype characteristics. We compared the differences in clinical and immune infiltration characteristics among these subtypes. Part 3: Identification of AD feature genes. We utilized the 13,582 genes to construct a gene co-expression network using the WGCNA algorithm. To assess whether a given co-expression module was related to AD, we correlated the module eigengene with various neuropathological aspects of AD (including Braak, NFTs, activity of α-, β-, and γ-secretases, and amyloid-beta 42), as well as the MMSE and the CDR. We identified a module that exhibits the strongest relevance to AD progression. The LASSO and RF models were subsequently constructed to analyze the candidate module genes, which were selected from the WGCNA results. Finally, the feature genes were identified by intersecting the genes selected through the two algorithms, leading to the development of a predictive model for AD. Part 4: Validation of AD feature genes. We further analyzed single-cell genome-wide transcriptome to explore cell type-specific AD-related dysregulation of AD feature genes selected by the integrative works of supervised (RF, LASSO) and unsupervised (WGCNA) algorithms. Furthermore, animal experiments were conducted to validate the AD feature genes

Due to technical limitations, most studies currently focus on only one or two necroptosis-associated genes (NRGs), while the complexity of AD progression involves numerous genes interacting in a coordinated manner. Necroptosis can be triggered by various stimuli, such as tumor necrosis factor (TNF), TNF-related apoptosis-inducing ligand (TRAIL), Fas ligand (FasL), interferon (IFN), LPS, viral DNA or RNA, DNA-damage agent and requires the kinase activity of receptor-interacting protein 1 (RIPK1) and RIPK3. And its execution involves ROS generation, calcium overload, the opening of the mitochondrial permeability transition pore, mitochondrial fission, inflammatory response and chromatinolysis. Hence, we gathered a total of 159 NRGs from KEGG database, a comprehensive database integrating information on various biological processes, and previously published reviews [10–12]. Based on NRGs expression levels, 667 AD patients were classified into three distinct subtypes by non-negative matrix factorization (NMF). Subsequently, we assessed differences in necroptosis scores, cognitive function, pathological characteristics, and immune cell infiltration among these subtypes. By employing Weighted Correlation Network Analysis (WGCNA) and machine learning algorithms (RF, LASSO), we identified eight feature genes of AD. We further validated the expression of these feature genes using bulk RNA-seq, single-nucleus RNA-seq, and in vivo experiments. The overall research strategy employed in this study is showed in Fig. 1, and the findings hold promising implications for advancing our understanding of the molecular pathogenesis of Alzheimer’s disease and identifying potential diagnostic markers.

## Methods

### Acquisition and pre-processing of AD datasets

We acquired AD datasets from the GEO database [13]. We downloaded a total of eight datasets as follows: GSE106241, GSE118553, GSE122063 [14], GSE132903 [15], GSE28146 [16], GSE48350 [17], GSE5281 [18], GSE8442201 (GSE84422 comprises datasets from three distinct platforms, with GSE8442201 being the dataset annotated by GPL570). The general information of datasets is available in Table. S1. To ensure data quality, the eight raw datasets were preprocessed by data filtering, background adjustment, log2 transformation, and normalization. In addition, the “Combat” algorithm was used to correct for batch effects between these eight datasets. The merged dataset consisted of a total of 1170 samples, including 667 AD samples and 503 normal samples.

### Single-cell sequencing analysis

We also downloaded the single-nucleus sequencing (snRNA-seq) dataset GSE157827 [19] and GSE174367 [20], containing 23 AD samples and 16 normal samples. The “Seurat” packages were employed to analyze the snRNA-seq dataset. Quality control measures were applied to remove potential doublets and low-quality cells. Cells were filtered out if nFeature < 300 or > 10,000 and nCount > 100,000, while cells with percent.mt > 10% were filtered out as well. Next, we performed dimensionality reduction and clustering and selected 3000 hypervariable genes. By combining with the elbow plot and selecting the inflection point and the PC with a smooth curve, we selected the first 30 dimensions for follow-up analysis and showed the dimension reduction effects of UMAP. Finally, we visualized the expression of the feature gene in different cell types using a violin diagram.

### Identification of necroptosis-related subtypes

According to the necroptosis pathway (hsa04217) in the KEGG database, we obtained 159 necroptosis-associated genes (NRGs) (https://www.genome.jp/dbget-bin/www_bget?pathway+hsa04217). Table S2 provides detailed genes information. On the basis of NRGs, we performed unsupervised NMF on the merged dataset [21].

### Clinical characteristics between subtypes

The gene set variation analysis (GSVA) analysis was performed to calculate the necroptosis score for each sample using NRGs. In order to assess the clinic and pathological characteristics between different subtypes, a pairwise comparison was conducted using Wilcoxon’s rank-sum test. This analysis encompassed the evaluation of necroptosis score, neuropathological hallmarks (Braak, NFT, α-, β-, and γ-secretases), age, as well as cognitive function (CDR). Aβ plaques generation owing to the sequential enzymatic cleavage of amyloid precursor protein by β-secretase and γ-secretase [22]. Braak represents the staging score for tau neurofibrillary tangles, where higher scores indicate a greater extent of tangle burden. Furthermore, the proportion of APOE4 alleles in different groups were also calculated.

### Immune infiltration characteristics of necroptosis-related subtypes

A variety of algorithms are utilized to evaluate the immune infiltration signature. The “IOBR” package utilized the following algorithms: EPIC [23], IPS [24], QUANTISEQ [25], CIBERSORT [26], TIMER [27], XCELL [28], MCP-Counter [28] and ESTIMATE [29]. These algorithms were used to evaluate the types and relative abundance of infiltrating immune cells. Additionally, the single-sample gene-set enrichment analysis (ssGSEA) was applied to calculate an enrichment score, which indicates the degree of coordinated up- or down-regulation of genes within a specific gene set in a single sample [30].

### Identification of hub genes in AD

We utilized the genes of 667 AD samples to construct a gene co-expression network by the WGCNA algorithm. First, we performed a scale-free topology standard to identify the optimal soft-threshold power. Second, we calculated a weighted adjacency matrix and converted it into a topological overlap matrix. Third, we employed hierarchical clustering and tree analysis to identify modules that consist of more than 50 genes. Each module was represented by an eigengene. To assess whether a given co-expression module was related to AD, we correlated the module eigengene with various neuropathological aspects of AD (including Braak stage, neurofibrillary tangles, activity of α-, β-, and γ-secretases, and amyloid-beta 42), as well as the Mini-Mental Status Examination (MMSE), the Clinical Dementia Rating Scale (CDR), and AD subtypes. Finally, the co-expressed genes were determined by calculating the module membership (MM) and gene significance (GS) of the genes in the selected modules.

### Functional enrichment analysis of hub genes

Bioconductor package was employed to perform Gene Ontology (GO) analysis on the hub genes of blue models.

### Screening the AD feature genes based on machine learning

Given poor AD neuropathological signatures of the cluster B and its notable enrichment in immune infiltration, we conducted an investigation into the module of cluster B. We developed two machine-learning algorithms: LASSO regression and RF algorithm. The LASSO regression algorithm is a well-established linear prediction method that relies on regression coefficients [31]. We conducted the R software’s “glmnet” packages to conduct LASSO analyses. The RF algorithm stands as one of the most widely recognized and accepted multi-class tree algorithm using the “randomforest” package. Finally, the feature genes were identified by the intersection of the genes from the two algorithms.

### Development and validation of the feature genes predictive model for AD

The merged dataset was randomly divided into a training set (70%, N = 819) and a testing set (30%, N = 351). Diagnostic nomogram of AD was constructed by fitting the feature genes into a binary logistic regression model with the training set. Predictive performance for the nomogram was assessed using calibration curves, decision curve analysis (DCA) and area under the receiving operating characteristic curve (AUC) values.

To further estimate the discriminatory capacity of the feature genes for patients with AD and controls, we used six validation datasets: GSE122063, GSE118553, GSE5281, GSE132903, GSE48350, and GSE28146 to plot the AUC by the “pROC” package.

### Animals

The P301S mouse, which carries the human tau gene with the P301S mutation, serves as a well-characterized mouse model for AD research. The P301S transgenic mice was a kind gift from Professor Gang Li [32]. Transgenic and nontransgenic mice were all littermates of P301S mice. We used 8-month-old P301S mice (male, n = 9) were used as an in vivo AD model and age-matched male C57BL/6 J mice (n = 9) were used as controls. During the experiment, all mice were housed under standard laboratory conditions and a light/dark cycle was maintained artificially of 12/12 h. All animal experiments were reviewed and approved by the Ethics Committee of Tongji Medical College, Huazhong University of Science and Technology.

### Quantitative reverse-transcription polymerase chain reaction

Half of the mice from each group were randomly chosen and anesthetized. The cortices of the mice were surgically removed. Total RNA was extracted using TRIzol reagent. The mRNA was reverse transcribed to cDNA using a reverse transcription kit (Takara, Japan). The cDNA, primers, and ChamQ SYBR qPCR Master Mix (Vazyme, China) were combined into a polymerase chain reaction (PCR) reaction plate and the mRNA levels of *CARTPT, KLHL35, NRN1, NT5DC3, PCYOX1L, RHOQ, SLC6A12, SLC38A2* were measured using StepOnePlus real-time PCR System. The primer sequences are provided in Table. S3 and all experiments were repeated thrice.

### Western blot analysis

The tissues were thoroughly homogenized in RIPA lysis buffer (Servicebio, G2002) utilizing the technique of ultrasonication to facilitate comprehensive extraction of total protein. Subsequently, the lysates were subjected to centrifugation, and the protein concentrations were determined employing the BCA Protein Assay Kit (Boster, AR0146). Equal quantities of protein (15 μg) from each sample were then segregated on an SDS-PAGE gel and subsequently transferred onto polyvinylidene fluoride (PVDF) membranes. These membranes were subsequently subjected to blocking with 5% skimmed milk for a duration of 1 h at ambient temperature. Following the requisite washing steps, the membranes were incubated overnight at 4 °C with the primary antibodies, namely the anti-RHOQ antibody (ABclonal, A19786). Following the incubation period, the membranes were washed thrice and incubated for 1 h at room temperature with the suitable secondary horseradish peroxidase (HRP)-conjugated antibody, specifically the goat anti-rabbit antibody (Abbkine, A21020). After a final round of washing, an enhanced chemiluminescence kit (Biosharp, BL520A) was employed for membrane staining, with the subsequent detection of bands accomplished using a gel imaging system (Syngene, United Kingdom).

### Immunohistochemistry

The first step was to cut the brain into 4-μm sections and mount them on slides. Afterward, the sections were deparaffinized in xylene and rehydrated in ethanol at graded concentrations, followed by water baths in citrate solution (pH = 6.0) for antigen retrieval and washed with PBS trice. Incubation occurred overnight at 4 °C with a 1:100 anti-CARTPT antibody (Abclonal, A18275) after blocking in 3% bovineserum albumin. The sections were then washed three times, incubated for 1 h with the appropriate secondary HRP- conjugated antibodie, and stained with 3,3′ diaminobenzidine. Afterwards, the slides were counterstained with Mayer’s hematoxylin (Absin, abs9215), and dehydrated. Eventually, the slides were covered slipped. The images were collected using an Olympus VS120 slice scanning system.

### Statistical analyses

Statistical analyses were implemented using R (version 4.2.0). Between-group comparisons were conducted with Wilcoxon test. A P-value of < 0.05 was considered statistically significant.

## Results

### NMF identifies three subtypes in the AD patients

**Fig. 2:**
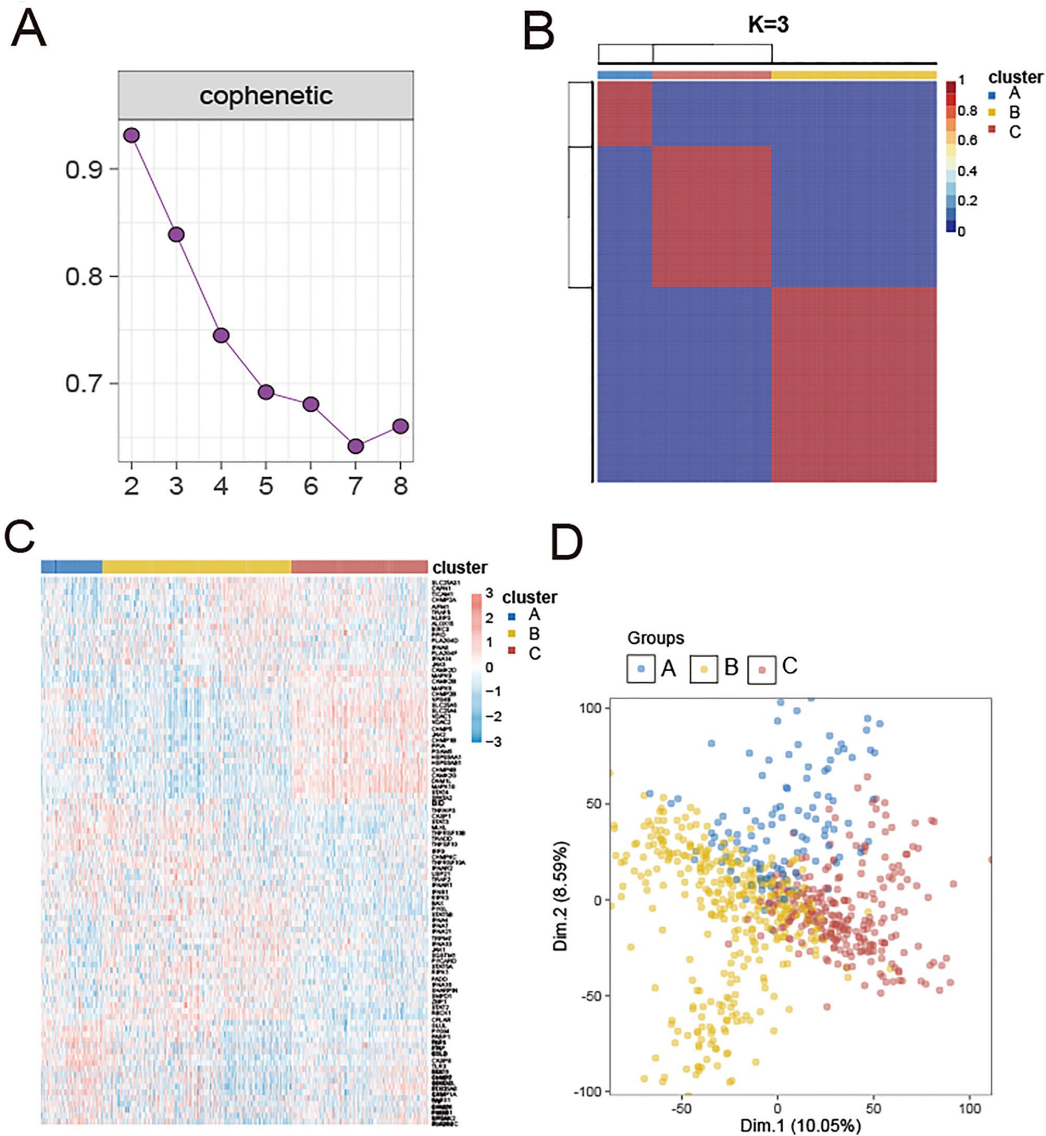
Identification of three necroptosis-related subtypes in ADs. **A** Cophenetic correlation coefficient of NMF When k = 2–8. **B** Consensus matrix heatmap when k = 3. Three subtypes were named as Cluster A, Cluster B, and Cluster C. **C** Necroptosis genes expression heatmap in three subtypes. Red indicates high expression. Blue indicates low expression. White indicates no difference. **D** The PCA plot of the samples from three different subtypes. The blue spots indicated the samples from cluster A. The yellow spots indicated the samples from cluster B, and the red spots indicated the samples from cluster C. PCA supported the classification of three AD subtypes

The research strategy employed in this study is visually represented in the flowchart (Fig. 1). As previously reported [33], no significant transcriptomes differences are observed among different anatomical locations within the brain (Fig. S1). Hence, in order to mitigate the data issues arising from excessive adjustment, we only performed the correction on batch effects. After removing the batch effects, datasets from different platforms grouped together (Fig. S2). Unsupervised NMF methods were performed to classify 667 AD samples into distinct subtypes according to the 159 NRGs. Cophenetic correlation coefficients were computed to ascertain the ideal value of “k”. After an evaluation process considering both the clinical characteristics and the Cophenetic value, we have determined that the optimal number of clusters is 3, denoted by the value “k = 3”. This decision was made to facilitate meaningful analysis (Fig. 2A, three subtypes were designated Cluster A, Cluster B, and Cluster C). When the value of “k” is set to 3, the consensus matrix heatmap still exhibits distinct and clear boundaries, indicating a stable and resilient clustering pattern for the samples (Fig. 2B). Thus, the AD patients were divided into three subtypes. The NRGs expression heatmap further confirmed significant discrepancies in the NRGs expression profiles between the three subtypes (Fig. 2C). Subsequently, to compare the overall transcriptional patterns of the different subtypes, the principal component analysis (PCA) was conducted. The result of PCA showed that the samples from the three subtypes were highly isolated from one another, which indicated distinct transcriptional profiles among these subtypes (Fig. 2D).

### Clinical characteristics between three necroptosis subtypes

**Fig. 3:**
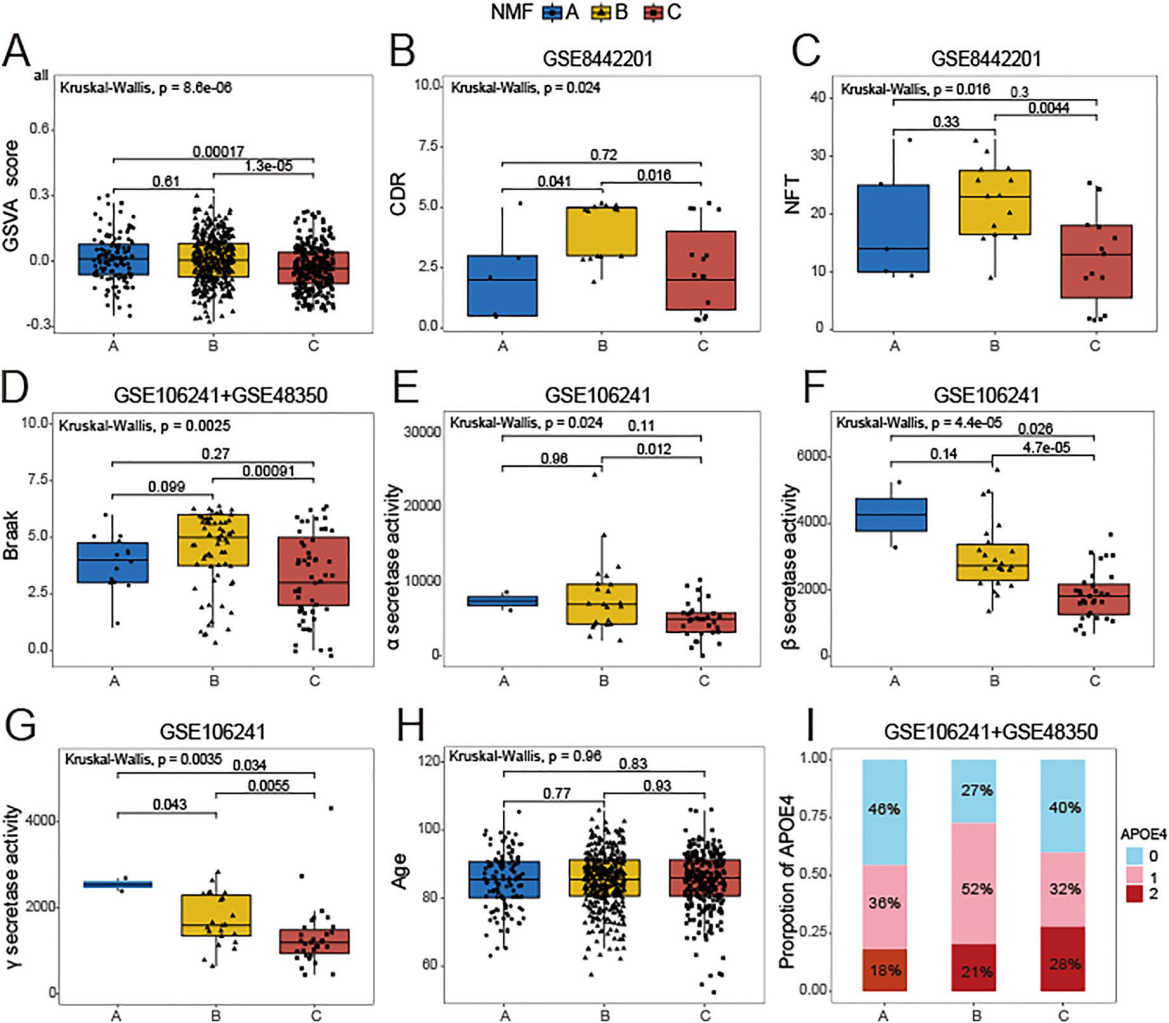
Clinical characteristics of necroptosis-related subtypes. Comparison of **A** necroptosis scores, **B** CDR, **C** NFT, **D** Braak, **E** α-secretase activity, **F** β-secretase activity, **G** γ-secretase activity, and **H** Age between three subgroups. **I** The proportion of APOE 4 alleles of each subgroup (0 indicates no APOE4 allele, 1 indicates one APOE4 allele, 2 indicates two APOE4 alleles.). The clinical data of **B**, **C** is from GSE8442201 (cluster A, n = 5; cluster B, n = 14; cluster C, n = 15). The clinical data of **D**, **I** is from GSE106241 and GSE48350(cluster A, n = 14; cluster B, n = 70; cluster C, n = 56). The clinical data of **E**–**G** is from GSE106241(cluster A, n = 3; cluster B, n = 26; cluster C, n = 31). The clinical data of **G**, **H**, **I** is from merged 8 datasets (cluster A, n = 119; cluster B, n = 359; cluster C, n = 259). CDR, clinical dementia rating score (higher scores representing worse functional status); Braak, tau neurofibrillary tangle staging score (higher scores represent greater extent of tangle burden); β-secretase and γ-secretase serve as the principal catalysts in the sequential enzymatic cleavage of amyloid precursor protein, leading to the generation of Aβ plaques

Upon comparing the levels of necroptosis score across the three subtypes, Cluster B exhibited the highest necroptosis score, followed by Cluster A, and ultimately Cluster C (Fig. 3A). In the evaluation of CDR, Cluster B displayed the worst functional status, with Cluster A ranking as the second worst, and Cluster C emerging as the most favorable (Fig. 3B). In addition, the NFT and Braak in cluster B were also highest than other subtypes (Fig. 3C, D). The activities of α-, β-, and γ-secretases are all highest in cluster A compared to other subtypes (Fig. 3E–G). Of particular significance was the pronounced increase observed in the activities of β- and γ- secretases. (Fig. 3F, G). With regard to age, no distinction was observed between the three subgroups (Fig. 3H). In all subgroups, the proportion of individuals possessing the APOE4 alleles exceeded that of individuals without the APOE4 alleles (Fig. 3I). The tissue origin and sex proportion of different subtypes were shown in Fig. S3.

### Immune infiltration signature of necroptosis-related subtypes

**Fig. 4:**
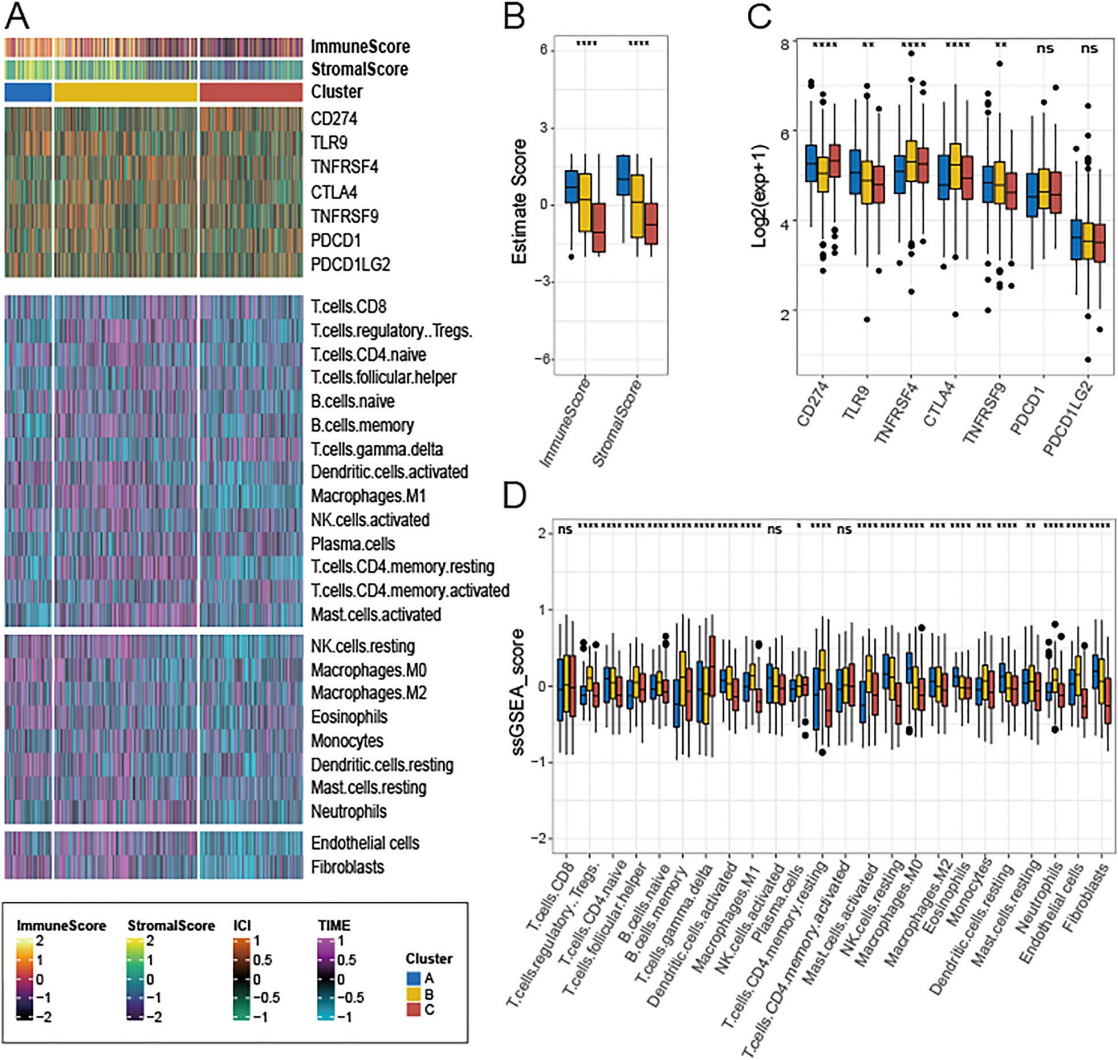
Immune signature of three subtypes. **A** Heatmap showing the immune infiltration landscape in three AD subgroups. Boxplots showing the differences of **B** immune and stromal score, **C** immune checkpoint targets, **D** infiltrated immune cells in three AD subgroups. (ns indicates no significance, *P < 0.05, **P < 0.01, ***P < 0.005, ****P < 0.0001)

We further explored the correlations between immune infiltrating levels. The heatmap of immune infiltration signature based on EPIC, IPS, QUANTISEQ, CIBERSORT, TIMER, XCELL, MCP-Counter and ESTIMATETIMER using the ssGSEA method is illustrated in Fig. S4 . The cluster A exhibiting higher values in both stromal and immune scores compared to other subtypes (P < 0.0001; Fig. 4B). We quantified the different infiltration degrees of 24 immune cell types and assessed the expression of immune checkpoints within the samples (Fig. 4A). We observed higher expression of multiple immune checkpoints in cluster B, which may be targets for immunotherapy, including TNFRSF4 and CTLA4 (Fig. 4C). Additionally, as shown in Fig. 4B, the proportion of 12 immune cells (Regulatory T-cells, T follicular helper cells, naive B cells, Memory B cells, M1 Macrophages, naive CD4 T cells, resting memory CD4 T cells, activated Mast cells, Monocytes, resting Mast cells, Neutrophils, Endothelial cells) were significant higher in cluster B compared to other subtypes (Fig. 4D).

### WGCNA to identify hub genes of AD

**Fig. 5:**
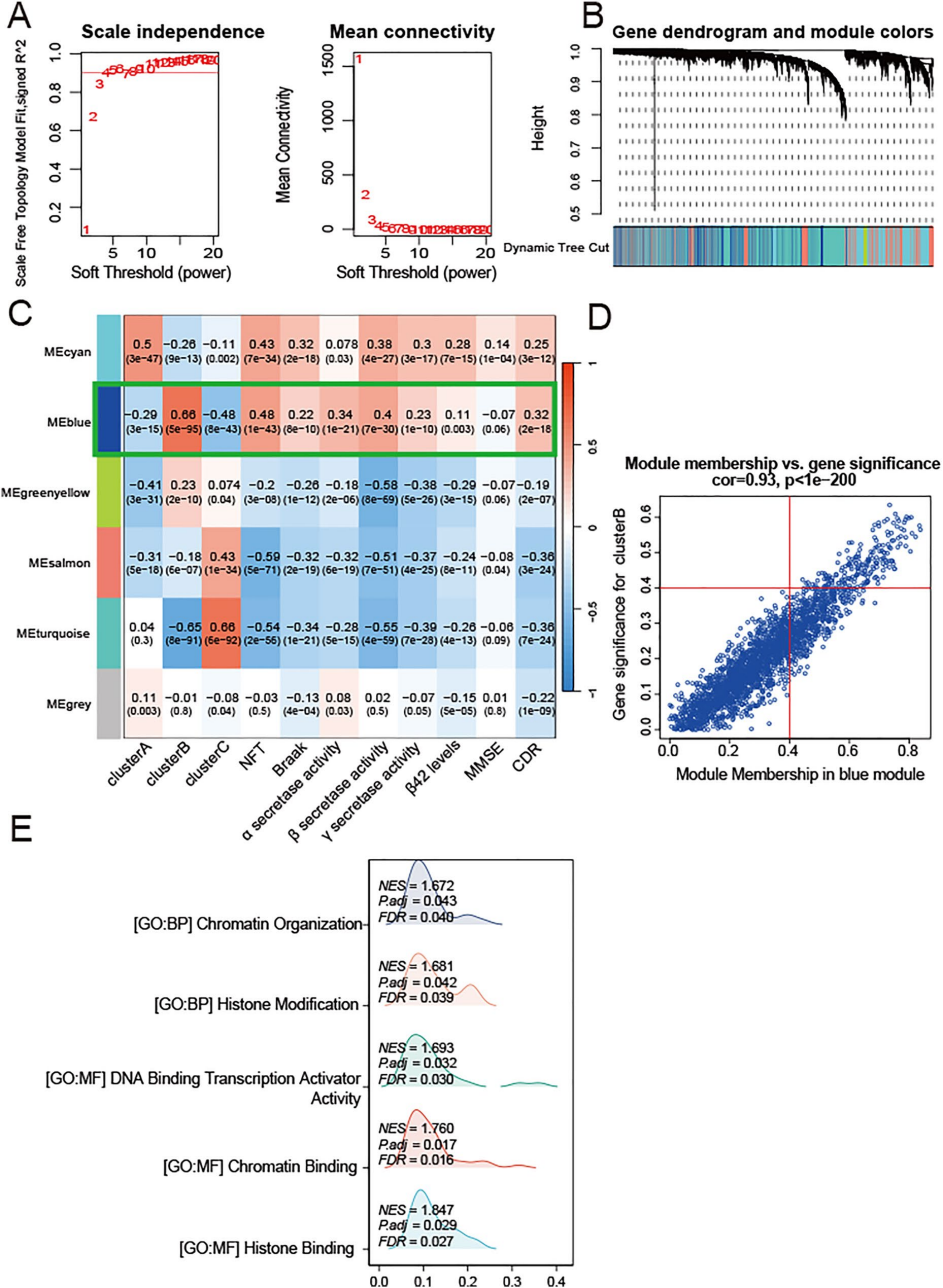
Identification of key module and functional enrichment analysis of hub genes. **A** Analysis of the scale index and the mean connectivity for various soft-threshold. **B** Hierarchical clustering dendrograms of co-expressed genes in modules. **C** Heatmaps of the correlation between Eigengene and clinical traits of AD. Strength of positive (red) or negative (blue) correlation is shown by two-color heatmap. The framed blue module was the key module most relevant to the Cluster B. The corresponding correlation and p value were shown in the cell (p value is enclosed within the brackets). **D** Scatter diagram showing the correlation of gene significance in blue module with gene significance. **E** Ridgeline plots showing the GO enrichment analysis of hub genes

We conducted WGCNA using the merged dataset to identify the module associated with poor AD progression. When the soft-threshold was 4, the scale-free network and connectivity exhibited maximum efficiency (Fig. 5A). Six gene modules were created using the hierarchical clustering algorithm (Fig. 5B). The blue module, which had 2367 genes, was the most closely associated with Cluster B (R = 0.66) as well as a series of AD-related high-risk indicators, including NFTs (R = 0.48), Braak (R = 0.22), alpha-secretase activity (R = 0.34), beta-secretase activity (R = 0.4), gamma-secretase activity (R = 0.23), amyloid-beta 42 (R = 0.11), MMSE (Minimum Mental State Examination, R = − 0.07) and CDR (R = 0.32) (Fig. 5C). Cluster B with high necroptosis scores had the worst neuropathology and cognitive function assessment. NFT and Braak (higher scores represent greater extent of tangle burden); MMSE, mini-mental status examination score (higher scores represent better cognitive function); CDR, clinical dementia rating score (higher scores representing worse functional status). Therefore, the blue module presented the strongest correlations with poor AD progression. We identified hub genes within the blue module using the selection criteria cor.MM > 0.4 and cor.GS > 0.4 (Fig. 5D). As a result, a total of 1153 hub genes were screened out. In addition, we performed GO enrichment analysis using the aforementioned hub genes (Fig. 5E). GO enrichment analysis revealed that these hub genes were predominantly enriched in chromatin organization, histone modification and DAN binding transcription activator activity. These results indicate the important functions of these genes.

### Selection of the AD feature genes

**Fig. 6:**
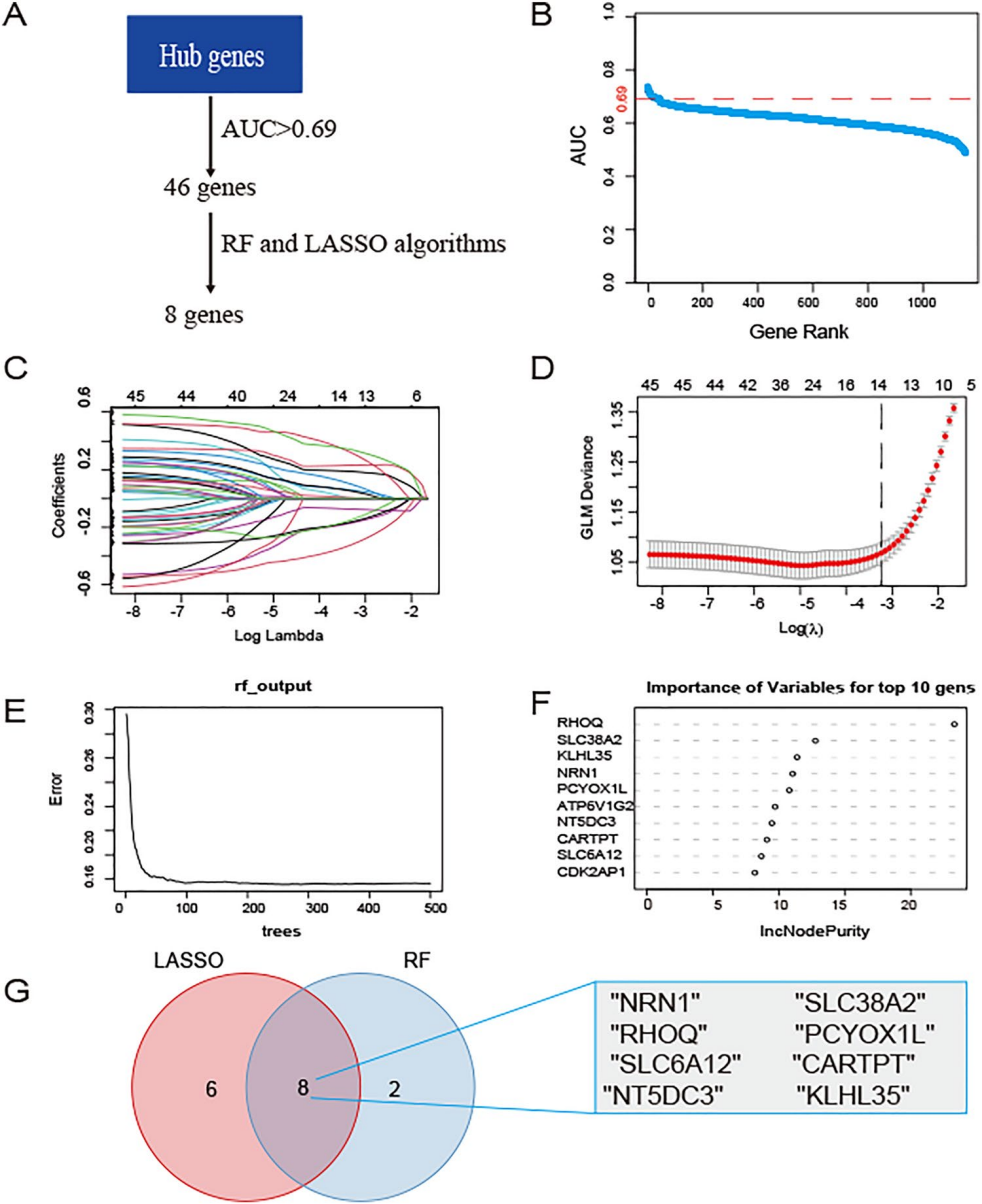
Screening the AD feature genes. **A** Flow chart of the process of selecting genes. The hub genes were identified from the blue module of the previous WGCNA step. **B** The AUC values of hub genes. A higher AUC value indicates a greater diagnostic value for AD. **C**, **D** LASSO regression to identified feature genes. **E**, **F** RF to identify the top 10 feature genes. **G** Venn diagram showing the feature genes intersected by two machine-learning algorithms

A ROC analysis was conducted on the expression levels of hub genes (identified within the blue module based on the selection criteria of cor.MM > 0.4 and cor.GS > 0.4) using the “pROC” R package to examine whether genes expression levels can distinguish between AD and normal patients, and the area under curve (AUC) was calculated. A higher AUC value indicates a greater diagnostic value for AD. The AUC values for hub genes were provided in Table. S4. A total of 46 genes had AUC values greater than 0.69 (Fig. 6 B). To further screen the feature genes predicting AD from these 46 genes, we performed LASSO and RF analyses. LASSO, a machine learning algorithm that combines variable selection and regularization, can enhance predictive accuracy [34]. On the other hand, RF is a predictive algorithm that does not impose restrictions on variable conditions, making it capable of providing predictions without apparent variations [35]. The intersection of the two results can serve as the candidate feature genes for diagnosis [36, 37]. Using the LASSO regression algorithm, we identified a subset of 14 feature genes (Fig. 6C, D). The RF algorithm revealed the top 10 feature genes (Fig. 6E, F). There were 8 overlapping genes among the LASSO and RF algorithms (*CARTPT, KLHL35, NRN1, NT5DC3, PCYOX1L, RHOQ, SLC6A12, SLC38A2*) (Fig. 6G).

### Development and validation of the feature genes predictive model for AD

**Fig. 7:**
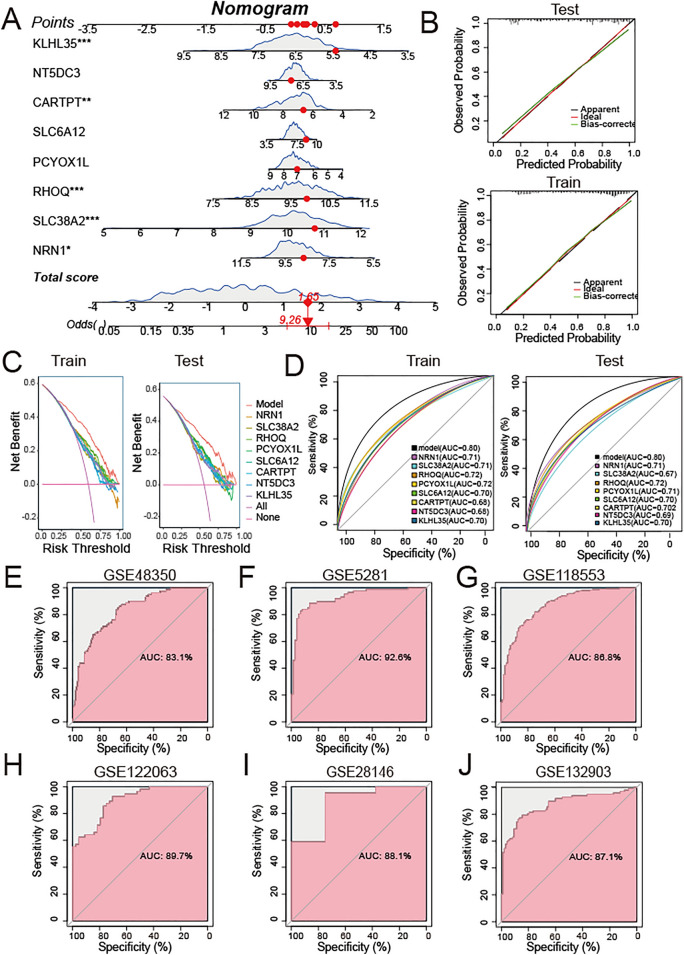
Development and validation of the feature genes predictive model for AD. **A** Nomogram of feature-genes predictive model for AD. **B** Calibration curve showing predicted performance of the nomogram in training and testing sets. **C** DCA showing the clinical benefits of the nomogram in training and testing sets. **D** ROC curves showing the diagnostic performance of feature genes in training and testing sets. **E**–**J** ROC curves showing the diagnostic performance of feature genes in 6 datasets including **E** GSE48350, **F** GSE5281, **G** GSE118553, **H** GSE122063,** I** GSE28146, **J** GSE132903. (*P < 0.05, **P < 0.01, ***P < 0.005, ****P < 0.0001)

A nomogram model was developed for AD diagnosis using 8 feature genes (*CARTPT, KLHL35, NRN1, NT5DC3, PCYOX1L, RHOQ, SLC6A12, SLC38A2*) (Fig. 7A). Calibration curves revealed a small error between the predicted and actual risk for AD, suggesting a high accuracy of the diagnostic model for predicting AD (Fig. 7B). DCA revealed that the “model” curve was higher than the curves representing “intervention for all,” “intervention for none,” and all single genes, suggesting that the patients may benefit from the diagnostic model, and the clinical benefit of the diagnostic model was higher compared with that of the single gene curve (Fig. 7C). The AUC of the diagnostic model was 0.8 in the train and test sets, indicating that it performed well in the discrimination between AD and normal samples (Fig. 7D). Furthermore, in six independent internal validation datasets, the five-gene diagnostic model yielded the AUC of 0.926 (GSE5281), 0.897 (GSE122063), 0.868 (GSE118553), 0.871 (GSE132903), 0.831 (GSE48350), 0.881 (GSE28146), respectively (Fig. 7E–L). These results indicate, to some extent, that the eight genes have an important role in AD pathogenesis.

### Validation of the feature genes expression

**Fig. 8:**
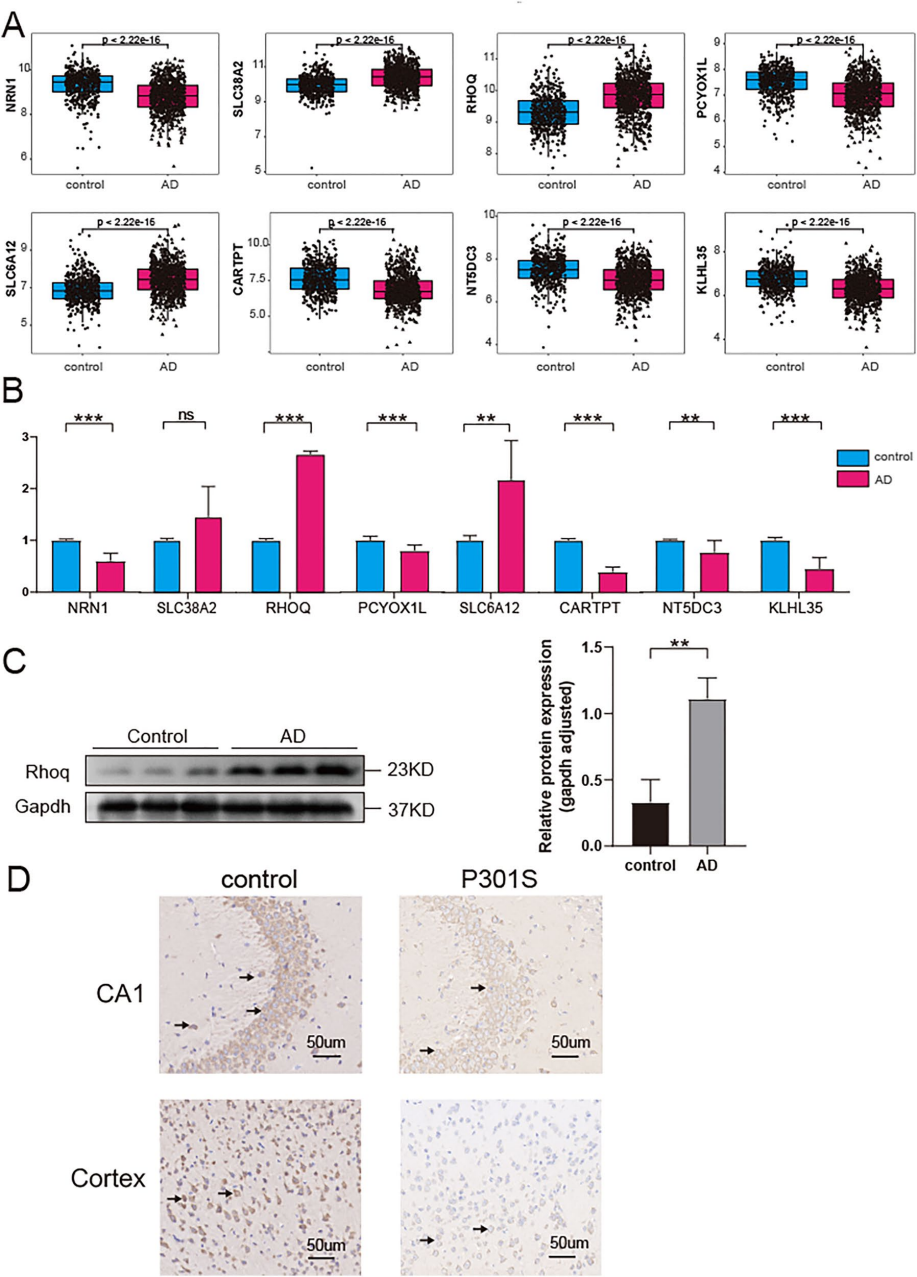
Validation of the eight feature genes expression between control and AD groups. **A** Expression levels of eight feature genes in the AD dataset. **B** The relative mRNA expression of eight genes was measured via qRT-PCR using mouse tissues (n = 3 in the control mice; n = 3 in the AD mice). **C** Western blotting of Rhoq. GAPDH was used as an internal control. (n = 3 in the control mice; n = 3 in the AD mice) **D** Immunohistochemical staining for CARTPT antibody in the brains of P301S and control mice. (scar bar: 50 µm). (*P < 0.05, **P < 0.01, ***P < 0.005, ****P < 0.0001)

The differential expressions of the feature genes were verified in the aforementioned merged dataset, which further demonstrated their diagnostic capacity for AD (Fig. 8A). In addition to the dataset, we conducted qRT-PCR experiments to further validate the expression of feature genes using tissues collected from AD mice or controls. Consistent with the bioinformatics analysis results, the expression of *RHOQ* and *SLC6A12* was significantly higher in AD mice compared with controls, whereas *CARTPT, KLHL35, NRN1, NT5DC3 and PCYOX1L* exhibited significant downregulation (Fig. 8B). To further confirm the reliability and accuracy of our bioinformatics analysis, *RHOQ* and *CARTPT* were selected for WB and IHC analyses (Fig. 8C, D). The results from WB and IHC analyses validated a significant upregulation of *RHOQ* in the brains of P301S mice, whereas *CARTPT* exhibited a downregulation in expression.

**Fig. 9:**
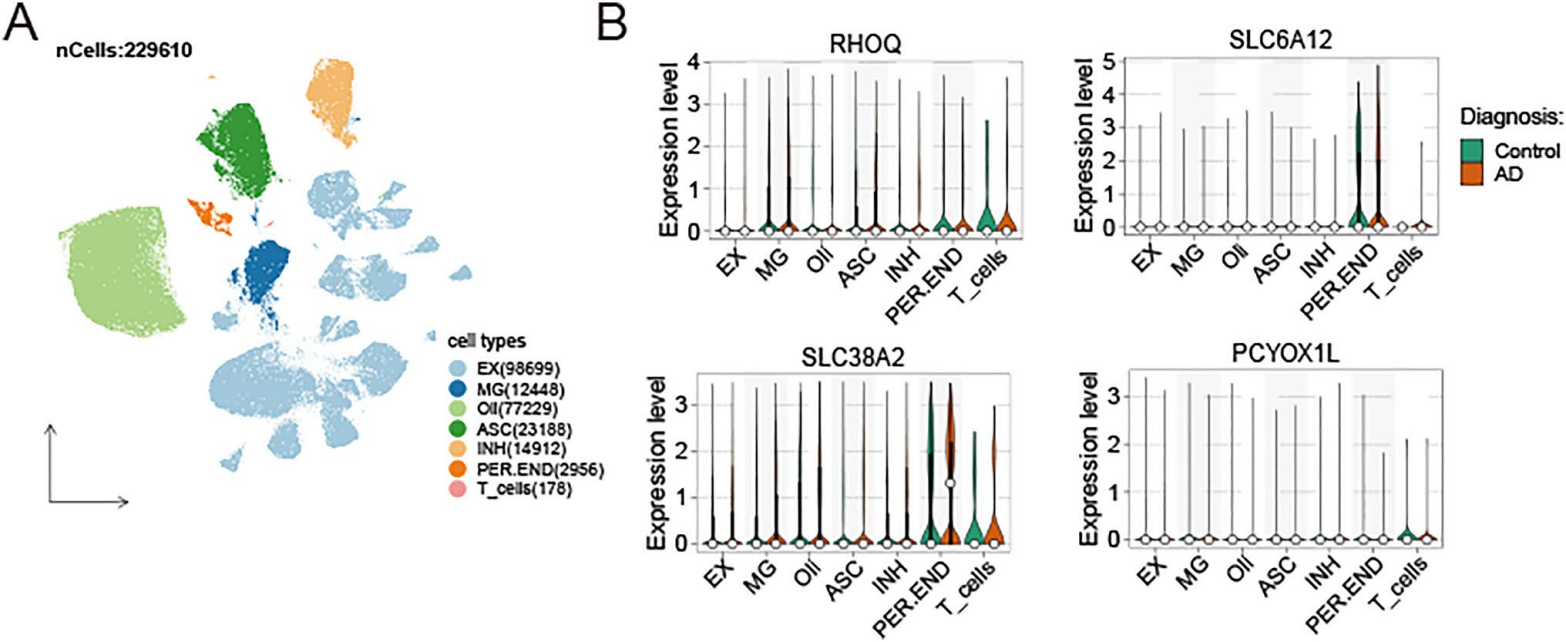
The expression of feature genes in the snRNA-seq data. **A** The landscape of diverse cell types is depicted through Uniform manifold approximation and projection (UMAP) visualizations. **B** The expression levels of RHOQ, SLC6A12, SLC38A2, and PCYOX1L in different cell types, split by control and AD samples. EX, excitatory neurons (98,699 nuclei); microglia (12,448 nuclei); Oli, oligodendrocytes (77,229 nuclei); ASC, astrocytes (23,188 nuclei); INH, inhibitory neurons (14,912 nuclei); PER-END, Pericytes/endothelial (2956 nuclei); T cells (178 nuclei)

We also downloaded the snRNA-seq dataset (GSE157827 and GSE174367) from the GEO database and conducted data quality control. Cells were filtered out if nFeature < 300 or > 10,000 and nCount > 100,000, while cells with percent.mt > 10% were filtered out as well. We performed dimensionality reduction and clustering and selected 3000 hypervariable genes. We employed uniform manifold approximation and projection (UMAP) dimensionality reduction and Leiden clustering to the batch-corrected transcriptomic datasets, screening different cell-type clusters within snRNA-seq (Fig. 9A). Finally, we visualized the expression of feature genes in different cell types by violin diagram. *RHOQ* and *SLC38A2* were annotated in all seven cell types, *SLC6A12* was by Pericytes/endothelial cells, *PCYOX1L* was only by T cells (Fig. 9B), *CARTPT, KLHL35, NRN1* and *NT5DC3* were hardly annotated (Fig. S5).

## Discussion

AD is a heterogeneous disease with intricate pathobiology [38, 39]. The presence of extracellular Aβ deposition as neuritic plaques and intracellular accumulation of hyperphosphorylated tau as NFTs remain the AD primary neuropathology. Regrettably, clinical trials targeting these plaques have mostly been unsuccessful so far [40]. Therefore, there is an urgent need to better understand the mechanisms behind AD in order to identify new biomarkers for early diagnosis, treatment and prognosis.

Neuronal death constitutes a significant hallmark of AD, playing a crucial role in its progression. However, the underlying mechanism responsible for neuronal cell death in AD remains unclear. In recent years, necroptosis has emerged as a promising therapeutic target, attracting notable attention as a potential avenue for intervention [41]. In this study, a total of 667 AD patients were classified into three molecular subtypes based on the necroptosis molecule expression levels. The significant differences were confirmed in clinicopathologic features and immune infiltration among distinct clustering subtypes. Cluster B with the highest necroptosis scores had the worst neuropathology and cognitive function assessment. Additionally, this cluster demonstrated the highest prevalence of individuals carrying the APOE gene. In contrast, Cluster C with the lowest necroptosis scores had the best assessment of neuropathology and cognitive function assessment. Our results suggest a significant correlation between necroptosis levels and AD.

Emerging evidence suggests that immune and inflammatory activation plays a critical role in the progression of AD [42, 43]. The accumulation of Aβ and NFTs leads to the impairment of synapses and the blood–brain barrier, which triggers activation of reactive microglia and astrocytes, as well as infiltration of certain peripheral immune cells into the brain [44]. Therefore, we employed the ssGSEA algorithm to further evaluate immune infiltration in AD necroptosis-related subtypes. The Cluster B with the highest necroptosis scores hold the most immune checkpoints, suggesting that the level of necroptosis is closely related to neuroimmune responses. The result also displayed a high relative abundance of 12 immune cell populations in cluster B, including naive and resting memory CD4 T cells, regulatory T cells, follicular helper T cells, naive and memory B cells, M1 Macrophages, resting and activated Mast, Monocytes, neutrophils, and endothelial cells, suggesting that these cells may be involved in the necroptosis mechanism of AD. It is possible that CD4 + T cells play a role in the pathogenesis of AD through their interaction with microglia, their involvement in immune mechanisms, and their role in facilitating amyloid clearance [45]. Depletion of NK cells in transgenic AD mouse models enhanced neurogenesis, reduced inflammation, and improved cognitive function [46]. Increased neutrophils have also been associated with cortical Aβ deposition and cognitive impairment [47].

We selected the gene modules with the highest correlation with Cluster B for analysis via WGCNA. Machine-learning algorithms have recently emerged as powerful tools for predicting biomarkers and providing insights into disease pathogenesis [48–50]. Therefore, based on the genes of blue module, eight hub genes were further screened from the blue modules by combining LR and LASSO machine learning methods. *CARTPT* encodes a neuropeptide involved in regulating appetite and satiety, with potential implications for connecting obesity and AD [51]. NRN1 plays a role in cell apoptosis, axonal regeneration, and synaptic maintenance [52–54]. Furthermore, a recent study conducted by Hurst et al. demonstrated the crucial role of NRN1 in promoting resilience of dendritic spines against Aβ in cultured neurons, unraveling its potential significance in mitigating the detrimental effects of AD [55]. SLC38A2 is a member of the amino acid transporter family, which acts as a sodium-dependent transporter for neutral α-amino acids [56]. It facilitates the movement of these amino acids across the blood–brain barrier and into neurons [57]. This process is crucial for supplying essential nutrients to support proper neuronal function. SLC6A 12 belongs to the solute carrier 6 (SLC6) family of sodium-dependent transporters and is responsible for regulating GABAergic neurotransmission to ensure homeostasis [58]. RHOQ, alternatively referred to as TC10, plays a crucial role in promoting neurite outgrowth through the regulation of membrane trafficking and cytoskeleton reorganization [59]. A recent study has revealed the indispensable requirement of PCYOX1L for the bactericidal activities of neutrophils [60]. NT5DC3, a member of the cytosolic 5′-nucleotidase II family, has exhibited a positive correlation between its expression level in a mouse model and the performance observed in reversal learning tasks [61]. The specific function of KLHL35 remains unclear, but recent investigations have demonstrated its association with DNA methylation and tumor mutation burden in cancers [62, 63]. The eight-gene predictive model showed a remarkable capacity for calibration and discrimination in predicting AD, suggesting that *CARTPT, KLHL35, NRN1, NT5DC3, PCYOX1L, RHOQ, SLC6A12* and *SLC38A2* have the potential to serve as diagnostic markers for AD. Furthermore, the qRT-PCR, WB and IHC experiments using brain tissues also confirmed the overexpression of *RHOQ* and *SLC6A12* and the downregulation of *CARTPT, KLHL35, NRN1, NT5DC3* and *PCYOX1L* in AD mice compared to controls. At present, apart from CARTPT and NRN1, the relationship between the remaining six genes and AD has not been investigated.

Despite the important findings of this study, there are still some limitations. First, the feature genes were only validated in animal experiment, with a lack of supporting vitro data. Second, *SLC38A2* displayed inconsistent results in the AD datasets and AD mice, possibly due to the small mice sample size. Finally, the underlying mechanisms through which necroptosis regulates immune infiltration in AD require further exploration in future studies.

## Conclusion

In conclusion, to the best of our knowledge, this study represents the first attempt to identify necroptosis subtypes in AD using NMF. Three AD subgroups with distinct necroptosis scores exhibited differences in clinical characteristics and immune infiltration, revealing the close correlation between AD, necroptosis, and immune infiltration. Our findings provide valuable insights into the heterogeneity of AD patients and lay a foundation for early intervention strategies. Furthermore, we successfully identified and validated five feature genes, including *CARTPT, KLHL35, NRN1, NT5DC3, PCYOX1L, RHOQ, SLC6A12, SLC38A2*. These genes exhibit close correlation with the prognosis of AD patients and have the potential to serve as biomarkers for the disease.

### Supplementary Information

Below is the link to the electronic supplementary material.

Supplementary file1 (DOCX 4552 KB)

Supplementary file2 (XLSX 60 KB)

## Data Availability

The datasets analysed during the current study are available in the GEO database (https://www.ncbi.nlm.nih.gov/geo/), openly available for free download.
