# Supplementary material for: Analysis and experimental validation of necroptosis-related molecular classification, immune signature and feature genes in Alzheimer’s disease
Source: Apoptosis. 2024 Mar 13;29(5-6):726–42. doi: 10.1007/s10495-024-01943-8 (PMC11055779; doi:10.1007/s10495-024-01943-8)
Supplement: Supplementary file 1 — Supplementary file1 (DOCX 4552 KB) [file 10495_2024_1943_MOESM1_ESM.docx]

**Supplementary Figures**

**
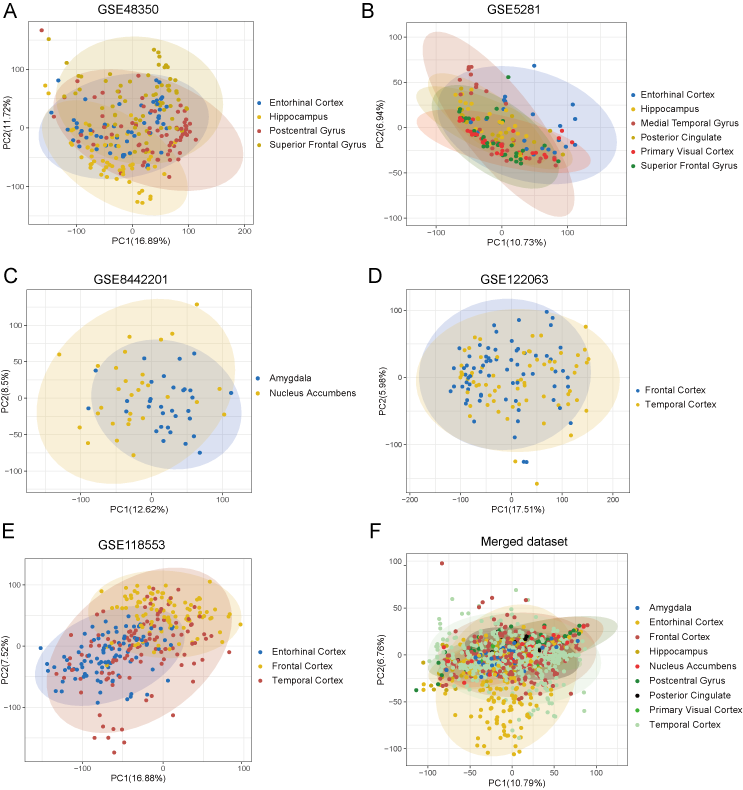
**

**Figure S1.** The PCA analysis among different brain regions. The imported samples data respectively comes from (**A**) GSE48350; (**B**) GSE5281; (**C**) GSE8442201; (**D**) GSE122063; (**E**) GSE118553; and (**F**) the merged dataset. The different colors of spots corresponded to samples originating from distinct anatomical locations. The PCA showed that the samples from the different brain regions were highly overlapping, suggesting similar transcriptional profiles among these brain regions.

**
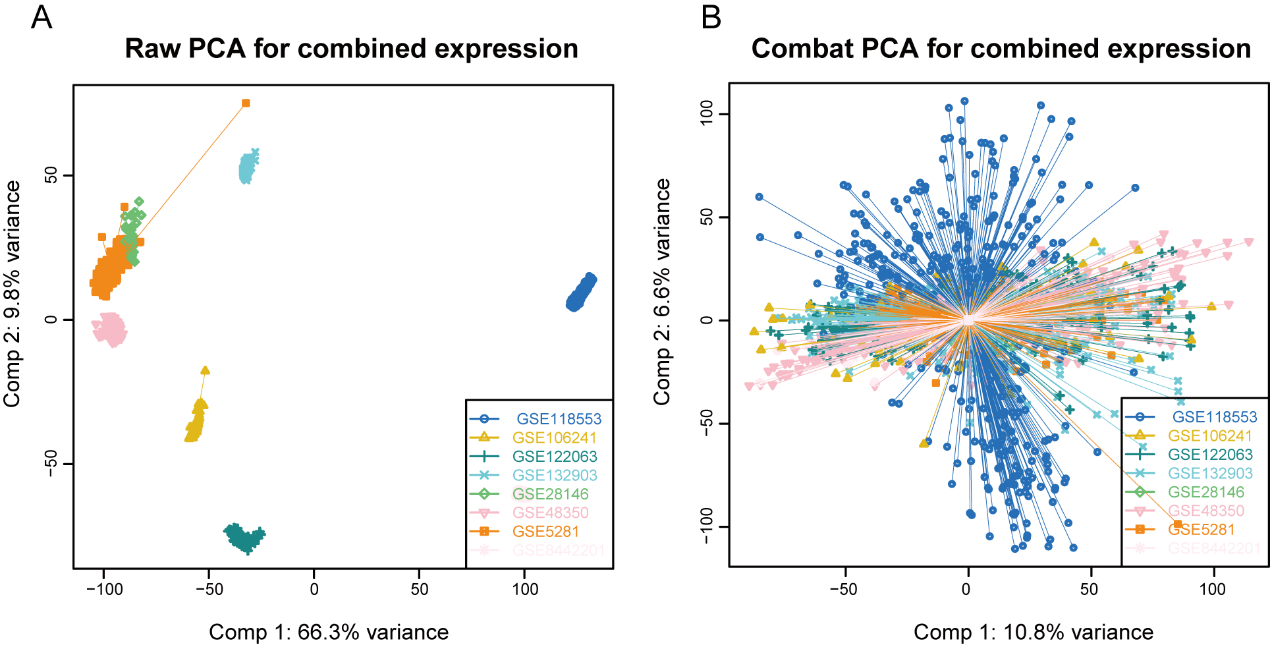
**

**Figure S2.** Principal component analysis (PCA) of 8 datasets before (A) and after (B) batch effect removal


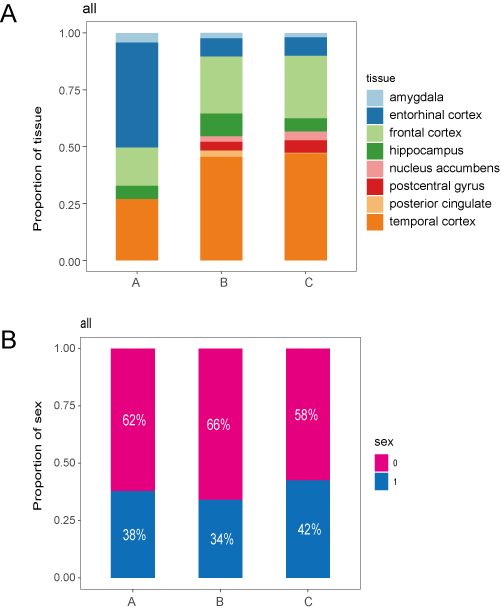


**Figure S3.** The proportion of tissues (**A**) and sex (**B**) in each subgroup.


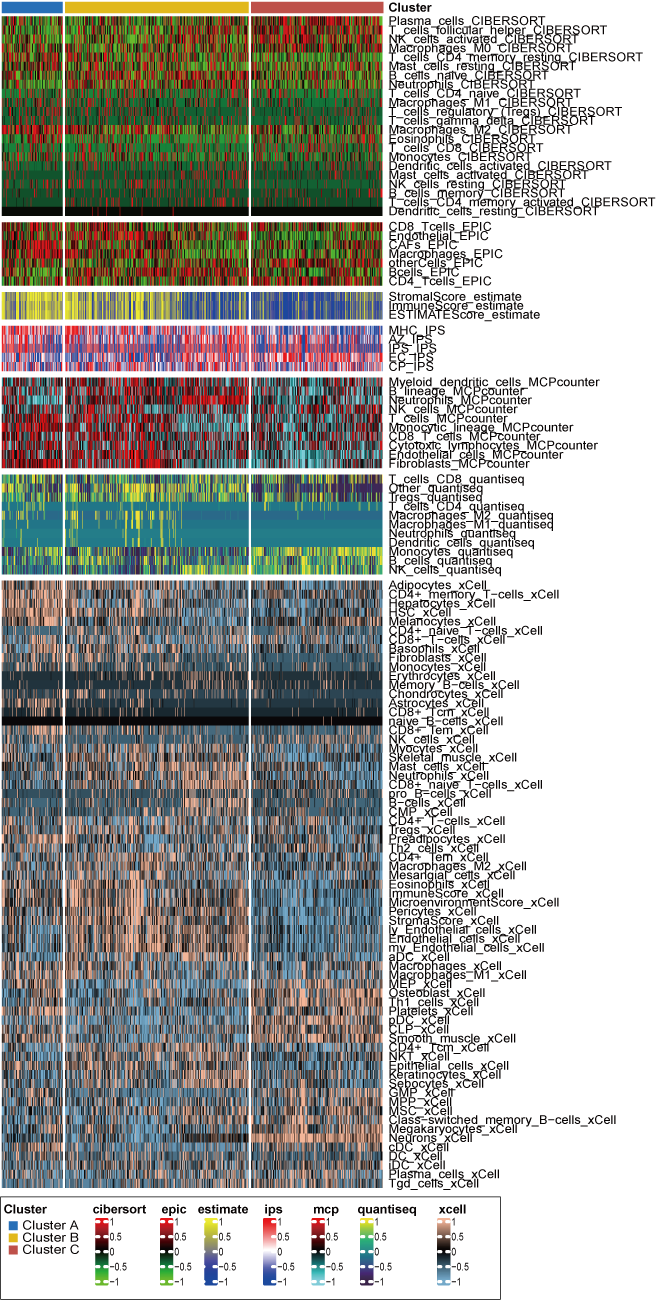


**Figure S4.** The heatmap of immune infiltration signature based on EPIC, IPS, QUANTISEQ, CIBERSORT, TIMER, XCELL, MCP-Counter and ESTIMATETIMER.


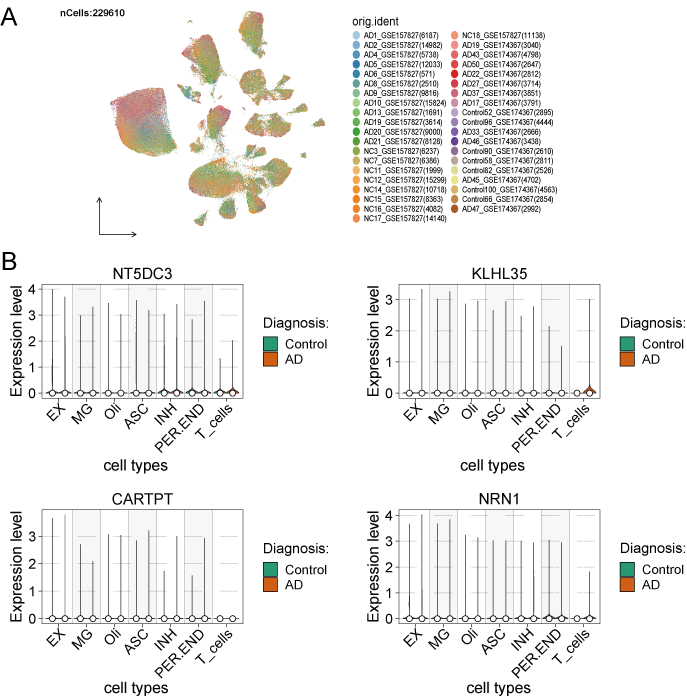


**Figure S5.**  The expression of feature genes in the snRNA-seq data. (**A**) UMAP plot shows cells from different sources. (**B**) The expression levels of CARTPT, KLHL35, NRN1, and NT5DC3 in different cell types, split by control and AD samples.
